# Use of Mechanical Turk as a MapReduce Framework for Macular OCT Segmentation

**DOI:** 10.1155/2016/6571547

**Published:** 2016-05-11

**Authors:** Aaron Y. Lee, Cecilia S. Lee, Pearse A. Keane, Adnan Tufail

**Affiliations:** ^1^Department of Ophthalmology, University of Washington, Seattle, WA 98104, USA; ^2^Medical Retina Service, Moorfields Eye Hospital NHS Foundation Trust, London EC1V 2PD, UK; ^3^Institute of Ophthalmology, University College London, London WC1E 6BT, UK; ^4^National Institute for Health Research Biomedical Research Centre for Ophthalmology, Moorfields Eye Hospital NHS Foundation Trust, London SE1 4TT, UK

## Abstract

*Purpose*. To evaluate the feasibility of using Mechanical Turk as a massively parallel platform to perform manual segmentations of macular spectral domain optical coherence tomography (SD-OCT) images using a MapReduce framework.* Methods.* A macular SD-OCT volume of 61 slice images was map-distributed to Amazon Mechanical Turk. Each Human Intelligence Task was set to $0.01 and required the user to draw five lines to outline the sublayers of the retinal OCT image after being shown example images. Each image was submitted twice for segmentation, and interrater reliability was calculated. The interface was created using custom HTML5 and JavaScript code, and data analysis was performed using R. An automated pipeline was developed to handle the map and reduce steps of the framework.* Results.* More than 93,500 data points were collected using this framework for the 61 images submitted. Pearson's correlation of interrater reliability was 0.995 (*p* < 0.0001) and coefficient of determination was 0.991. The cost of segmenting the macular volume was $1.21. A total of 22 individual Mechanical Turk users provided segmentations, each completing an average of 5.5 HITs. Each HIT was completed in an average of 4.43 minutes.* Conclusions.* Amazon Mechanical Turk provides a cost-effective, scalable, high-availability infrastructure for manual segmentation of OCT images.

## 1. Introduction

Crowdsourcing is a relatively novel technique involving the distribution of work to a large group of people, typically through online frameworks [1]. It allows the subdivision of tedious tasks into discrete tasks that can be completed individually. Amazon Mechanical Turk is the largest and most popular of the online crowdsourcing systems [2]. In this system, simple Human Intelligence Tasks (HITs) are submitted to online untrained users for a small compensation. Recently in computer science, the MapReduce programming model has caused a paradigm shift in the way that large data sets are distributed in parallel within a computing cluster [3]. Notably Google used the MapReduce framework to regenerate their index of the Internet, and the MapReduce framework has become popularized as a generic framework to solve big data problems in multicore cluster systems. In this study, our goal was to utilize human intelligence as a MapReduce framework for the segmentation of a macular optical coherence tomography (OCT) volume [4–6].

OCT is an important noninvasive diagnostic tool in the field of ophthalmology [6] and in the management of age-related macular degeneration (AMD), the commonest cause of blindness in the developed world [7, 8]. OCT measurements such as retinal thickness, subretinal fluid, and pigment epithelial detachment are important parameters in the diagnosis and monitoring of various retinal diseases [9, 10] and are thus integral in both large-scale clinical trials and routine clinical practice [11]. However, automated measurements provided by the OCT software result in frequent errors in quantifying critical parameters such as macular thickness and volume [12, 13]. Errors of retinal boundary detection and thickness measurements have been reported as high as 92% in segmentation performed by the Stratus OCT system (Carl Zeiss Meditec, Germany) [14]. Although spectral domain OCT (SD-OCT) is expected to produce more accurate measurements with higher resolution and less artifacts, the segmentation errors continue to be a significant problem in measuring macular thickness, particularly in eyes with pathology [15–17].

There has been increasing interest in ways to overcome automated segmentation errors. Publicly available image analysis software, OCTOR (Doheny Image Reading Center, Los Angeles), quantifies OCT-derived parameters after a trained OCT grader delineates the retinal boundaries of interest manually. The software calculates the distance in pixels between two manually drawn layers. Then using the dimensions of the B-scan image, the data is converted into a thickness measurement [18]. Even though OCTOR is less subject to segmentation errors, it is time-consuming and impractical for use in large-scale clinical trials.

**Figure 1:**
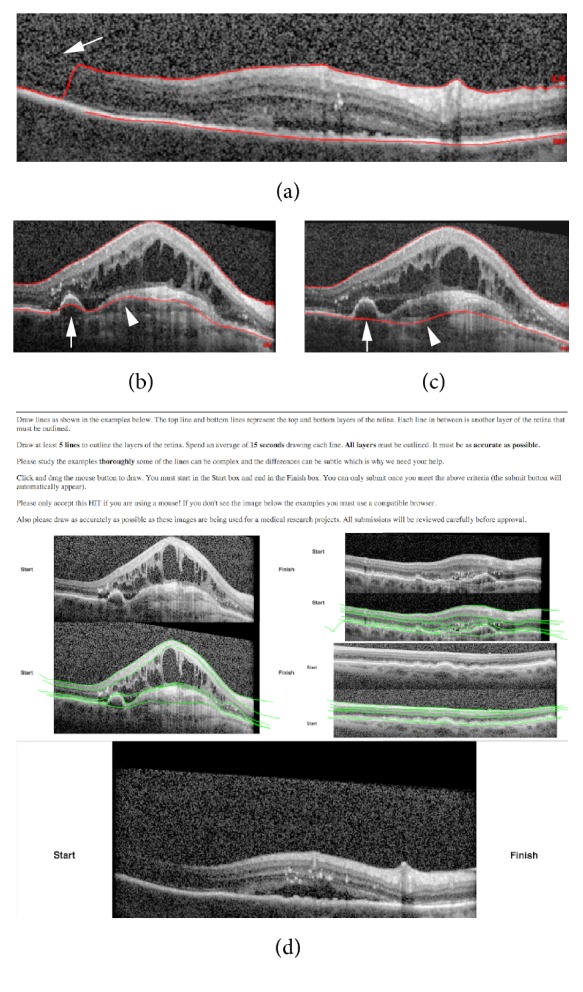
Examples of incorrect segmentations by automated software and user-interface for Mechanical Turk. Panel (a) is an example of a macula SD-OCT image with missing information (arrow) causing a sudden jump in the identification of the Internal-Limiting Membrane (ILM) by automated software included with Heidelberg Spectralis. Panels (b and c) show two similar macular OCT images with different automated segmentations caused by pigment epithelial detachment (arrow) and subretinal fibrosis (arrowhead). Panel (d) is a screenshot of web-based user-interface submitted to Amazon Mechanical Turk for manual segmentations.

Automated segmentations have been attempted using dual-scale gradient or intensity information. Then the edges of the boundary were optimized using a shortest path search method [19]. Statistical models have been utilized for a more reliable automatic segmentation system [20]. Retinal layers have been segmented using seven features extracted from the OCT data with a random forest classifier [21]. Despite these achievements in the field of automated segmentations, macular OCT images with complex subretinal pathology, intraretinal/subretinal fluid, or low signal to noise ratio continue to pose a challenge for computer vision (Figures 1(a)–1(c)).

Amazon Mechanical Turk and other modalities of crowdsourcing have been previously used in medical applications and demonstrated high level of accuracy in diagnostic accuracy [22–24]. In ophthalmology, retinal fundus photographs have been recently analyzed and showed an accuracy level at least comparable to automated programs and some trained graders [25]. To our knowledge, it has not been used to attain segmentations in macular OCT images with complex pathology. We sought to achieve highly reliable segmentation by designing a system for distributed OCT segmentation over a scalable, human based infrastructure and to show proof of concept results.

## 2. Materials and Methods

Patient identifiers were stripped out completely and pseudoanonymized, and on this basis and for retrospective use of anonymized data in the UK formal ethics committee review is not required. However, consent was still obtained from all patients in this study to use their OCT images for research. This study was conducted in accordance with the Declaration of Helsinki and the United Kingdom's Data Protection Act.

A total of 61 individual macular SD-OCT images were taken using a commercially available SD-OCT device (Spectralis, Heidelberg Engineering, Heidelberg, Germany) as part of routine medical care for AMD. The images were extracted using commercially provided software (Heyex DICOM Interface, Heidelberg Engineering, Heidelberg, Germany), and no image manipulation was performed.

A custom web-based user-interface was created with Hyper Text Markup Language 5 (HTML5) and JavaScript to allow Mechanical Turk users to directly draw on the images through their web browser (Figure 1(d)). This interface gave each user an example image of segmentation by an expert retina trained physician. The JavaScript interface allowed capture of the mouse input to draw segmentation lines on the provided OCT image and captured timing data as the user drew segmentation lines. Each user was instructed to draw 5 lines to segment the provided image and they were required to spend at least an average of 15 seconds per line before they were allowed to submit their work. No image enlargement or zoom was allowed and users were given 3 example segmentations provided by a trained OCT grader using the same system. Each image was created as a separate HIT and the reward was set to $0.01 (USD). Mechanical Turk users were required to have a prior approval rate of 80% before being allowed to participate in these HITs. In addition to the lines drawn, data was collected on the time spent drawing each line segment and time to completion of segmentation, and each image was submitted twice for segmentation.

After all segmentations were performed, the data was collected and image processing was performed to enhance the accuracy of the manual segmentations. This automated analysis pipeline used adjustments based on finding the consistently highest contrast value within 5 pixels of where the segmentation line was drawn. If there were no improved changes detected, then data from the original manual segmentation was used. Automated quality control heuristics were implemented to ensure that no two segmentations from the same user of the same image crossed paths. The reduction step of combining consensus segmentations of the same image was classified using a linear correlation heuristic, and these segmentation data were used to calculate interrater reliability. The final reduction step was utilized to recreate a three-dimensional segmented model of the retina. Custom Ruby and R code was created to automate the creation, submission, collection, image processing, and data analysis. All custom software is available upon request.

## 3. Results

The automated analysis pipeline, using a MapReduce framework, was able to create, submit, collect, collate, process, and analyze a total of over 92,500 data points from the 61 macular OCT images that were manually segmented twice over Amazon Mechanical Turk. Time of submission of the 122 HITs to completion of all tasks was 3 days with greater than 75% of HITs finished within the first 24 hours. A total of 22 individual Mechanical Turk users provided segmentations each completing an average of 5.5 HITs.

**Figure 2:**
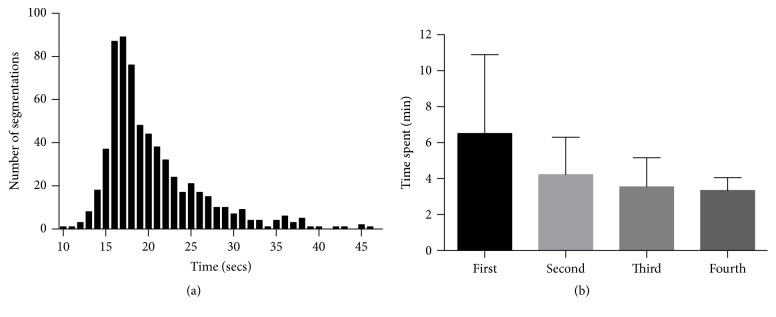
Temporal data of segmentations. Panel (a) shows a histogram of the time spent for each segmentation line. Average was 20.40 seconds with a range of 10.01 to 46.22 seconds. Panel (b) shows the decreasing trend in total time spent in minutes segmenting one SD-OCT image in a subset of users who segmented 4 or more times (7 out of 22 users). Error bars are standard deviation.

Each HIT was completed on average of 4.43 minutes (range: 1.83–24.45 minutes) with each segmentation line completed on average of 20.40 seconds (Figure 2(a)). In a subset of users who had segmented four or more HITs, we noted that there was a trend in decreasing time to completion of the task (Figure 2(b)). A total of 646 segmentations were collected, and an average of 5.30 segmentations per macular OCT was provided (range: 5 to 7). The total cost of segmentations of all images was $1.22 (USD).

**Figure 3:**
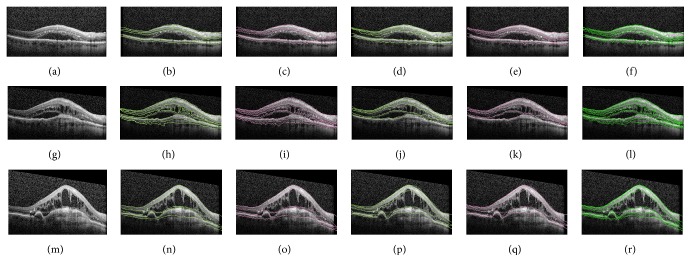
Representative segmentations by Mechanical Turk based manual segmentations with contrast based enhancements. Panels (a, g, m) show three raw SD-OCT macular images. Panels (b, d, h, j, n, p) demonstrate each image segmented by two different people on Mechanical Turk. Manual segmentations are shown as green lines. Panels (c, e, i, k, o, q) show local contrast based enhancement of manual segmentations as magenta lines. Panels (f, l, r) are the final consensus segmentations (green lines) after combining segmentations.

**Figure 4:**
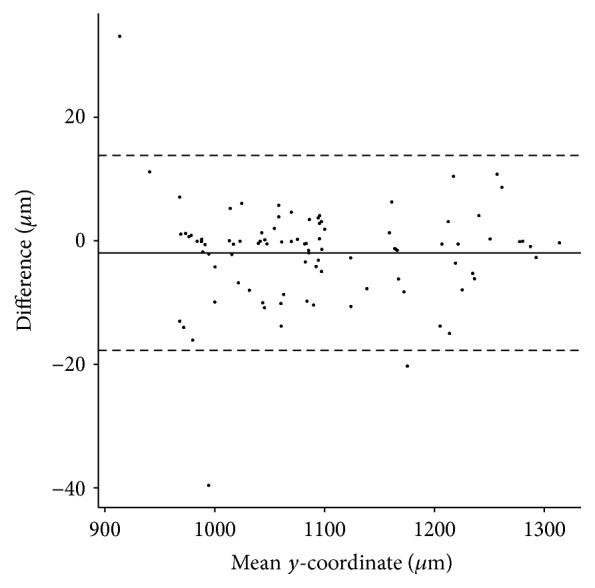
Bland-Altman plot showing agreement between segmentations. Consensus segmentations of the same image between two independent Mechanical Turk users were used to determine interrater reliability. The average *y*-coordinate value in microns for each consensus line was used and the Bland-Altman plot was created.

Representative segmentations with the associated image processing are shown in Figure 3. All slices from both manual segmentation and the combined final segmentations are shown in the Supplementary Materials (see Supplementary Material available online at http://dx.doi.org/10.1155/2016/6571547). Pearson's correlation of interrater reliability was 0.995 (*p* < 0.0001) and coefficient of determination was 0.991. A Bland-Altman plot was calculated to estimate interrater agreement based on the consensus segmentation lines (Figure 4).

## 4. Discussion

OCT is a critical tool in clinical practice for ophthalmology, and objective, quantitative OCT parameters have the potential of guiding clinical practice and establishing new endpoints for clinical trials. Automated segmentation approaches have traditionally suffered in the setting of complex retinal pathology such as pigment epithelial detachments, subretinal fibrosis, or intraretinal and subretinal fluid. Indeed the automated segmentation that is provided with the commercial device used in this study failed in many situations (Figures 1(a)–1(c)). With the advent of Mechanical Turk and programming APIs, automating simple human vision tasks through a MapReduce framework has become not only feasible but also cost-effective. The advantages of utilizing manual segmentations using human vision include the ability to complete areas of macular OCT where there is poor signal to noise ratio (Figure 3(a)) or complex pathology (Figure 3(c)).

Next steps of this study would be to compare the accuracy of the Mechanical Turk based segmentation to the ones performed by trained experts. Using the segmentation lines performed by trained experts as the gold standard, we will plan to evaluate the correlation between the accuracy and the time spent by the users, previous experience of the users, and any learning effect by repetitive performance of the same users.

Limitations of this approach stem from the lack of professionally trained OCT readers and the lack of knowledge or training of the Mechanical Turk users. Future analysis pipelines may include an expert validation step, which reviews the consensus segmentations and decides whether to accept or to reject the submitted segmentations, which then could be resubmitted for another round of segmentation. In addition, future, large-scale studies will be necessary to assess the external validity of this system by submitting macular OCT images for segmentation by expert graders versus Mechanical Turk.

Large data sets are becoming increasingly common with today's clinical studies and multicenter trials. Rapid, reliable, cost-effective methods of interpreting large data will be crucial in the future. Crowdsourcing in OCT segmentation has the potential of minimizing the errors seen in automated segmentation system with less time and cost than manual segmentations performed in reading centers. Additional ways to improve this tool such as more effective training of the users, preselection of qualified users, or creating an automated system based on users' initial segmentation would be important areas to be investigated. Implementation of our current method in the RISE/RIDE study, for example, where 759 patients received monthly OCT imaging, would cost approximately $273.24 per study month for a standard 18-slice macular OCT.

This study has applied a novel proof of concept of applying manual segmentation of OCT images in a distributed way to nonexpert graders. The retinas with various pathologies provide challenge to currently available automated segmentation systems. Mechanical Turk provides a cost-effective, scalable, high-availability infrastructure for manual segmentation of OCT images of the type which are difficult for automated algorithms to handle. The resulting images can be recombined for high-resolution 3D analysis. This approach may be applied to the analysis of high volumes of OCT images in clinical studies or training of future automated segmentation algorithms.

## Supplementary Material

Segmentations by Mechanical Turk based manual segmentations with contrast based enhancements. Each row represents a unique macular B scan image. The first column shows segmentations (green lines) which were performed by a different Mechanical Turk user from the second column. The third column represents the consensus segmentations after local contrast based enhancements.
