# Supplementary figures and images for: Use of Mechanical Turk as a MapReduce Framework for Macular OCT Segmentation
Source: J Ophthalmol. 2016 May 11;2016:6571547. doi: 10.1155/2016/6571547 (PMC4879255; doi:10.1155/2016/6571547)

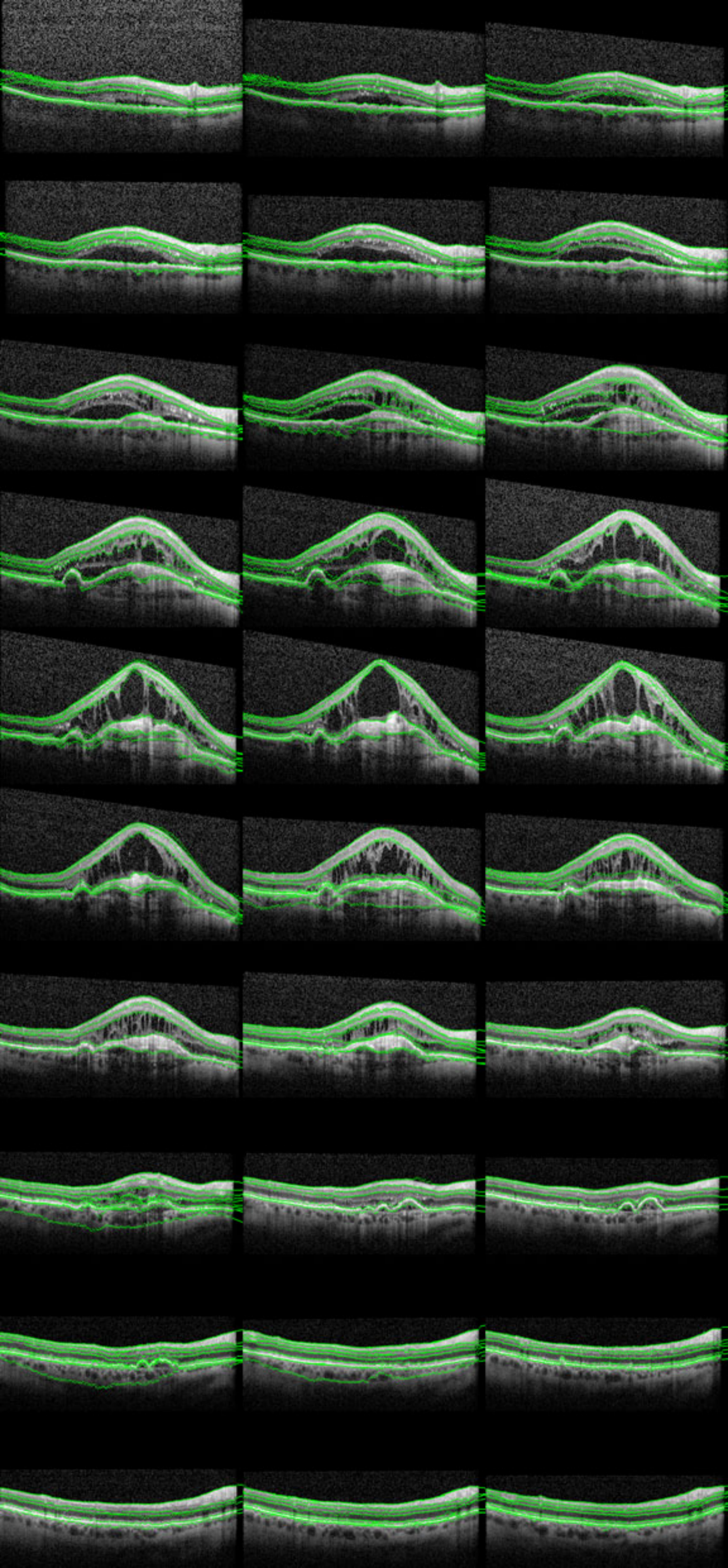

Supplement: Supplementary file 1 — Segmentations by Mechanical Turk based manual segmentations with contrast based enhancements. Each row represents a unique macular B scan image. The first column shows segmentations (green lines) which were performed by a different Mechanical Turk user from the second column. The third column represents the consensus segmentations after local contrast based enhancements. [file 6571547.f1.pdf]
